# Supplementary material for: Structural connectivity-based segmentation of the human entorhinal cortex
Source: Neuroimage. 2021 Dec 15;245:118723. doi: 10.1016/j.neuroimage.2021.118723 (PMC8756143; doi:10.1016/j.neuroimage.2021.118723)
Supplement: Supplementary file 2 [file mmc2.docx]

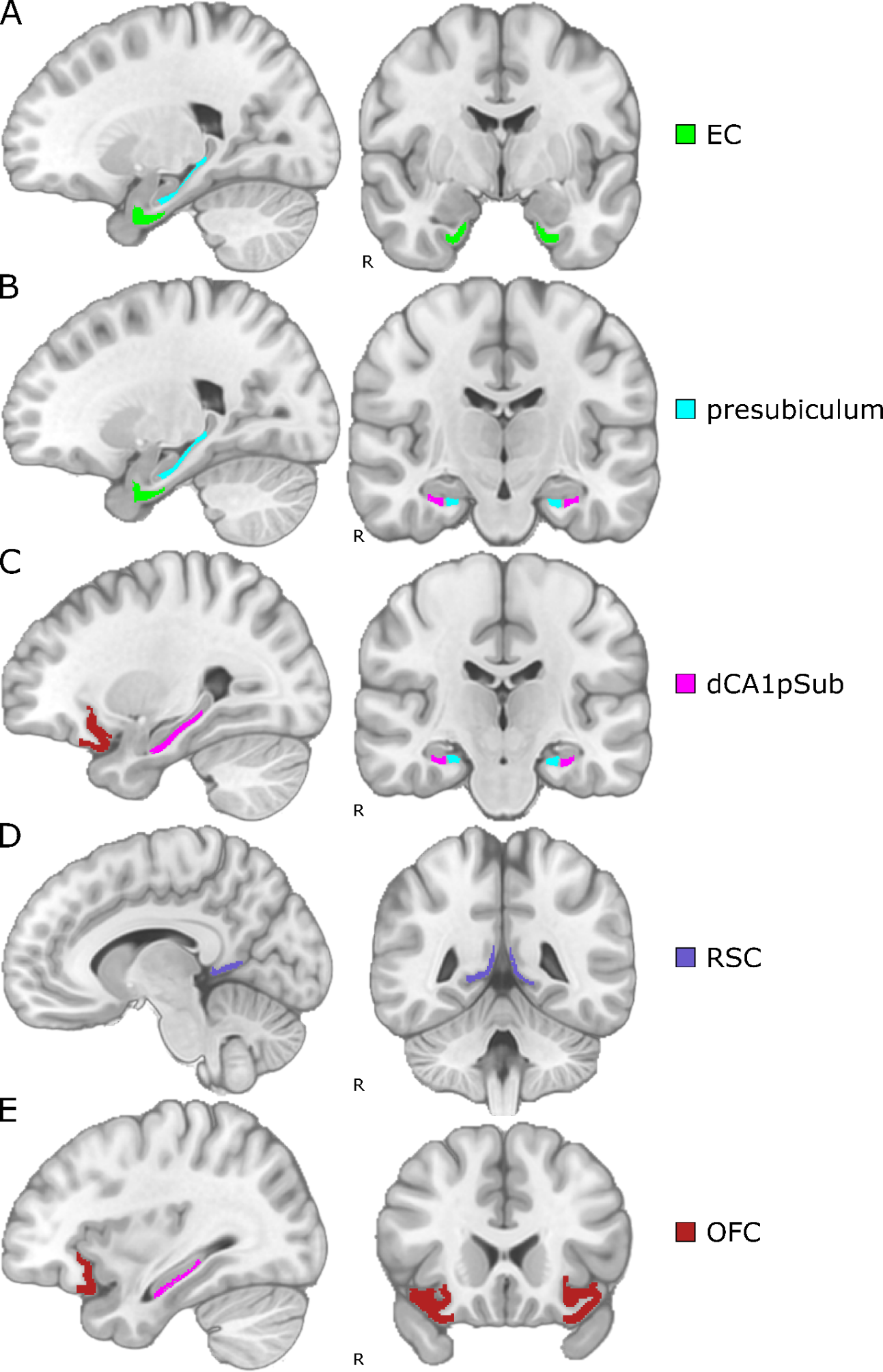


**Supplementary Figure 1: ROIs used for DTI analysis.** All ROIs are shown on a representative sagittal (left) and a coronal (right) slice in MNI space, with “R” denoting the right side of the brain. **A:** EC (green), **B:** presubiculum (light blue), **C:** dCA1pSub (pink), **D:** RSC (purple), **E:** OFC (dark red).


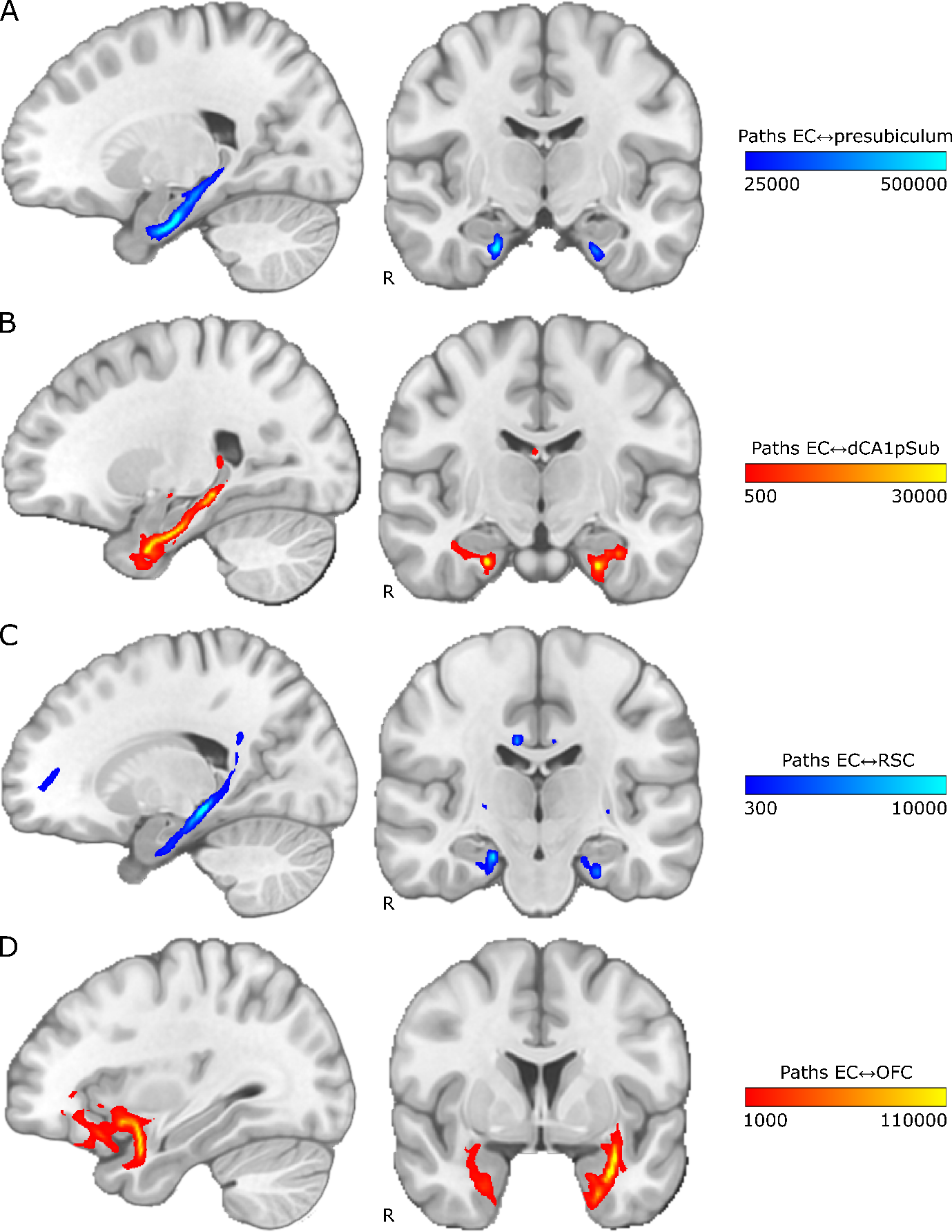


**Supplementary Figure 2:** **Connectivity paths between EC and presubiculum, dCA1pSub, RSC and OFC for one example participant.** The paths are shown on sagittal (left) and coronal (right) slices in MNI space, with “R” denoting the right side of the brain. The colormap intensity represents the number of probabilistic paths running through that voxel. **A:** Paths between EC and presubiculum, **B:** Paths between EC and dCA1pSub, **C:** Paths between EC and RSC, **D:** Paths between EC and OFC.


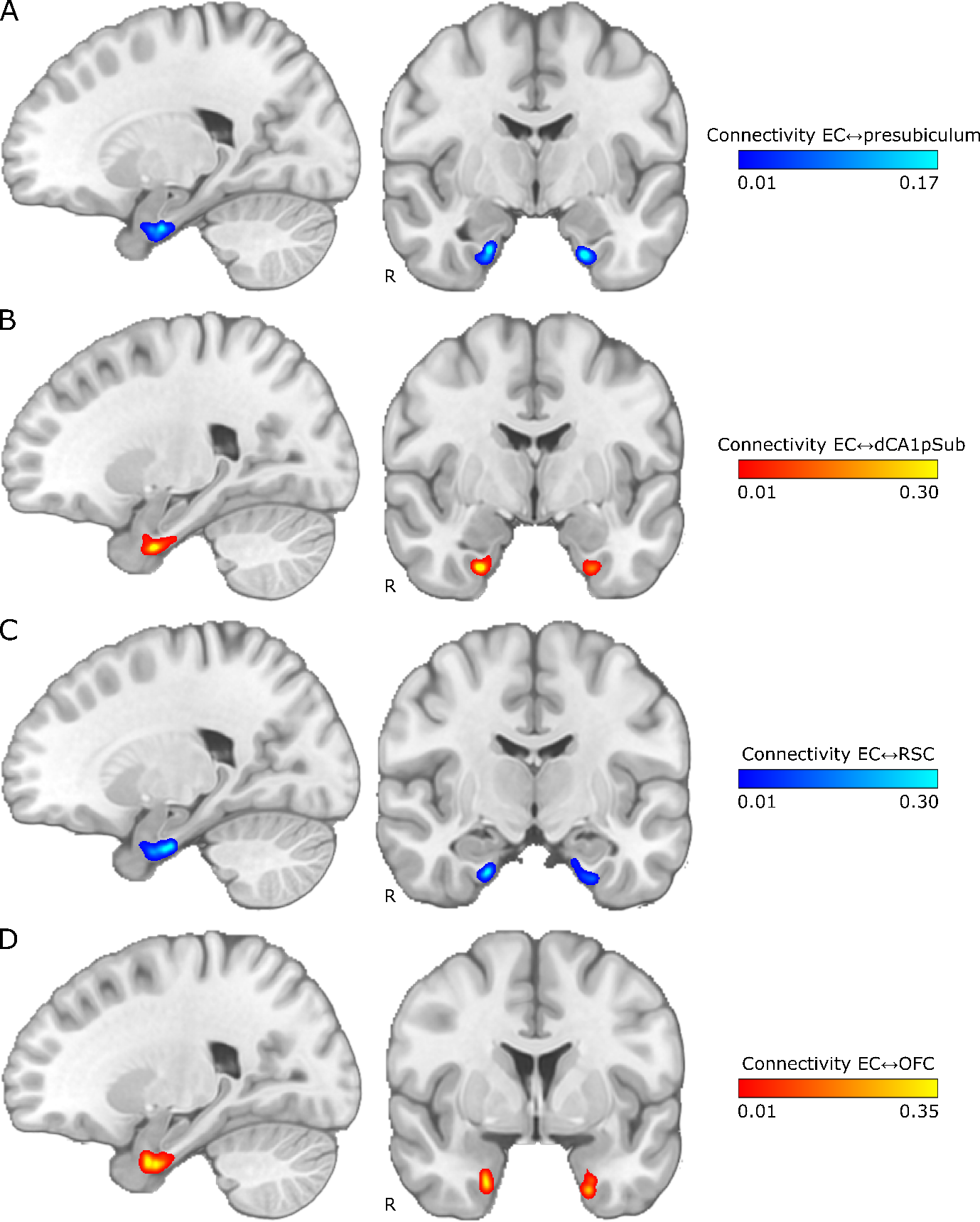


**Supplementary Figure 3:** **Maps of EC connectivity with presubiculum, dCA1pSub, RSC and OFC for one example participant.** The maps are shown on sagittal (left) and coronal (right) slices in MNI space, with “R” denoting the right side of the brain. The colormap intensity represents the fraction of paths seeded from that EC voxel that reached the other ROI. **A:** EC connectivity with presubiculum, **B:** EC connectivity with dCA1pSub, **C:** EC connectivity with RSC, **D:** EC connectivity with OFC.


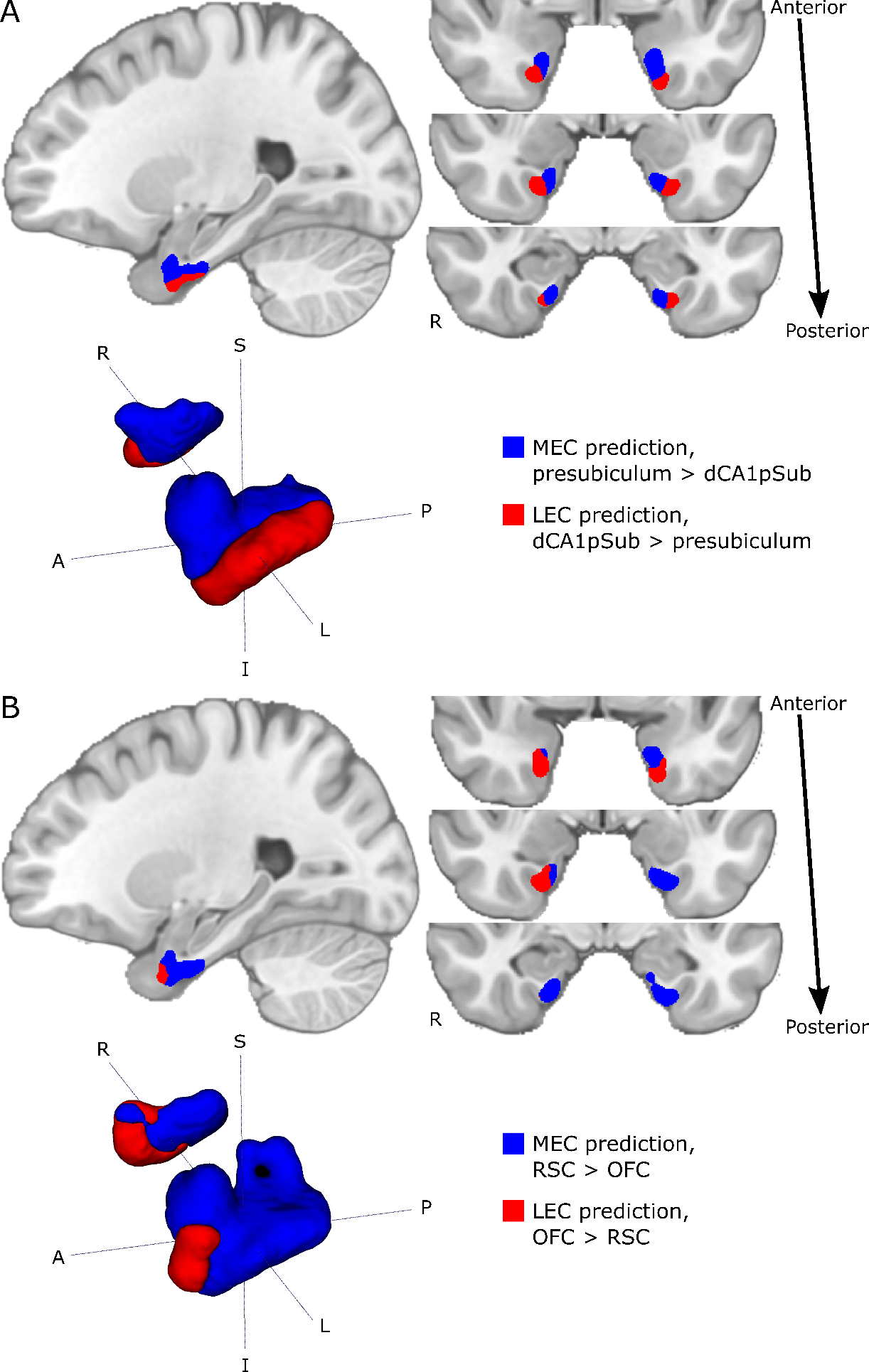


**Supplementary Figure 4:** **Segmentations of MEC and LEC from different approaches for one example participant.** The results are shown on sagittal (top left) and coronal (top right) slices and 3D-rendered (bottom left) in MNI space. The MEC and LEC predictions are shown in blue and red, respectively. **A:** MEC and LEC prediction based on connectivity with presubiculum vs. dCA1pSub, **B:** MEC and LEC prediction based on connectivity with RSC vs. OFC. S = superior, I = inferior, A = anterior, P = posterior, R = right, L = left.


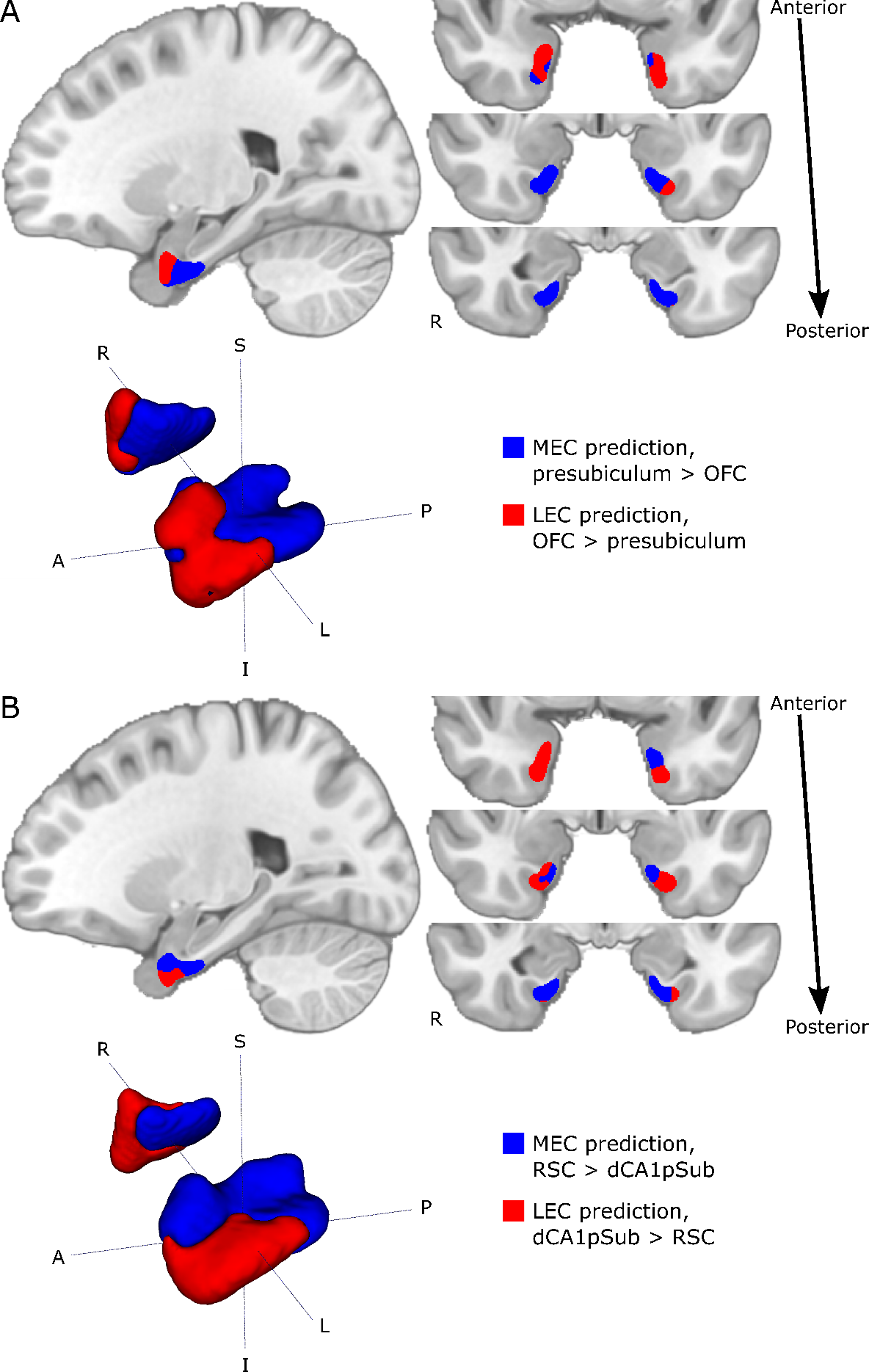


**Supplementary Figure 5:** **Group segmentations of MEC and LEC from different approaches.** The results are shown on sagittal (top left) and coronal (top right) slices and 3D-rendered (bottom left) in MNI space. The MEC and LEC predictions are shown in blue and red, respectively. **A:** MEC and LEC prediction based on connectivity with presubiculum vs. OFC, **B:** MEC and LEC prediction based on connectivity with RSC vs. dCA1pSub. S = superior, I = inferior, A = anterior, P = posterior, R = right, L = left.


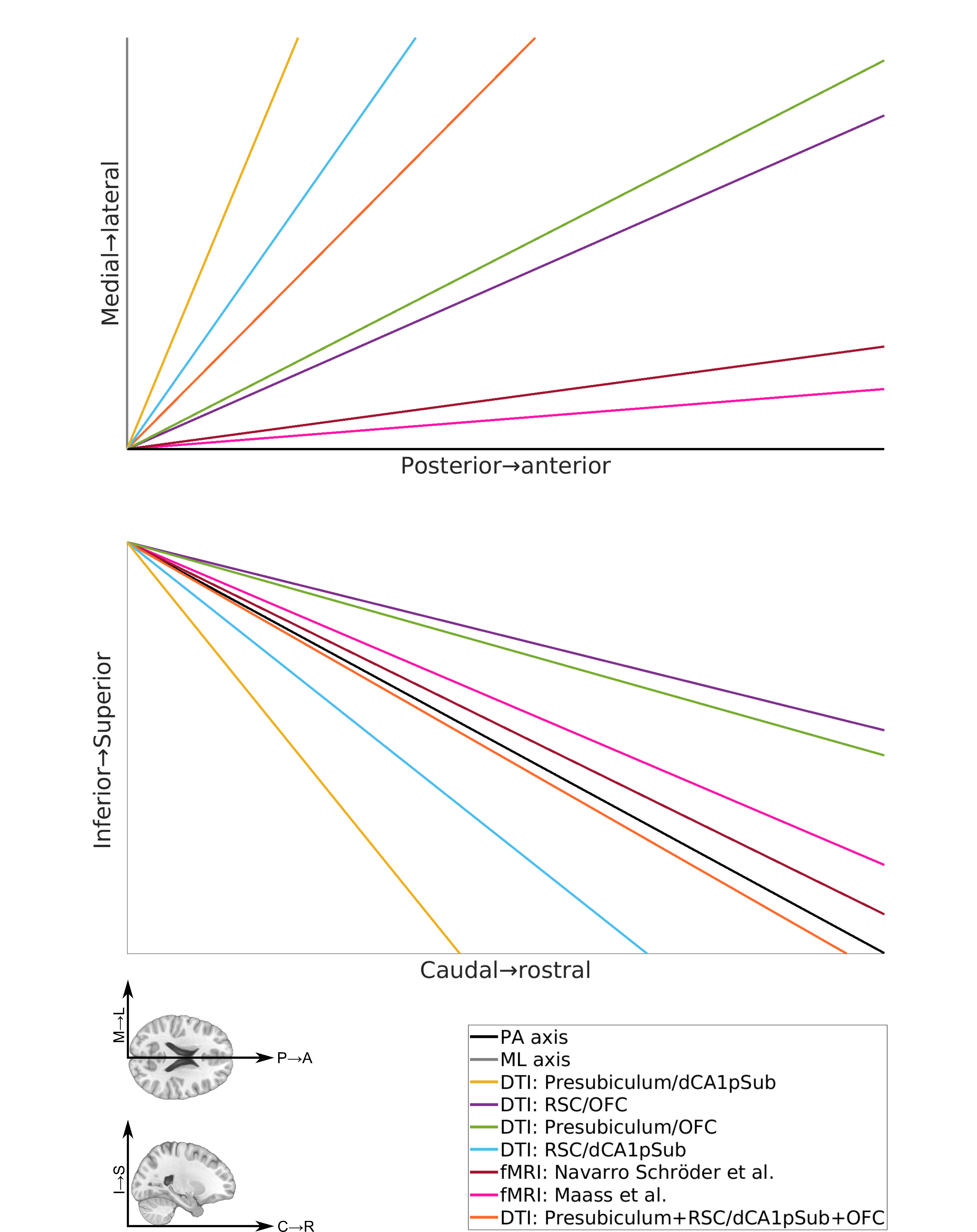


**Supplementary Figure 6:** **Center of gravity vectors between MEC-LEC segmentations in the axial (top) and sagittal (bottom) planes, showing the angles between the orientation of the MEC-LEC border and the pure PA and ML axes in the left hemisphere.** The PA axis vector is shown in black, the ML axis vector is shown in grey (visible in the axial view only), and the colors of the MEC-LEC vectors for all the different segmentation approaches are explained in the legend box on the bottom right. On the bottom left, illustrations of the anatomical directions of the vector plots are shown (M = medial, L = lateral, P = posterior, A = anterior, I = inferior, S = superior, C = caudal, R = rostral). The posterior-anterior axis is defined along the long axis of the hippocampus in the sagittal plane. Note that the length of the vectors shown here does not correspond to the real distance between the MEC and LEC centers of gravity. The origin of the vectors corresponds to the MEC center of gravity, which are not the true MEC locations for the different segmentation approaches, but shifted in space so that all vectors originate in the same point.
